# Association between the *TAP1* gene polymorphisms and recurrent respiratory papillomatosis in patients from Western Mexico: A pilot study

**DOI:** 10.1002/jcla.23712

**Published:** 2021-01-28

**Authors:** Jaime Palomares‐Marin, Luis Humberto Govea‐Camacho, Vania Araujo‐Caballero, Gerardo Cazarez‐Navarro, Sergio Yair Rodriguez‐Preciado, Enrique Ortiz‐Hernandez, Erika Martinez‐Lopez, Jose Francisco Muñoz‐Valle, Ivan Isidro Hernandez‐Cañaveral

**Affiliations:** ^1^ Departamento de Microbiología y Patología Centro Universitario de Ciencias de la Salud Universidad de Guadalajara Guadalajara México; ^2^ Servicio de Otorrinolaringología Cabeza y uello Centro Médico Nacional de Occidente IMSS Guadalajara México; ^3^ Servicio de Otorrinolaringología Hospital Civil de Guadalajara Fray Antonio Alcalde Guadalajara México; ^4^ Departamento de Biología Molecular y Genómica Centro Universitario de Ciencias de la Salud Universidad de Guadalajara Guadalajara México; ^5^ Instituto de Investigación en Ciencias Biomédicas Centro Universitario de Ciencias de la Salud Universidad de Guadalajara Guadalajara Mexico

**Keywords:** HPV, larynx, pediatric respiratory papillomatosis, recurrent respiratory papillomatosis, *TAP1*

## Abstract

**Background:**

Recurrent respiratory papillomatosis (RRP) is a respiratory tract disease that affects children and adults and is characterized by the recurrent proliferation of multiple papillomas. The etiologic agent is the human papillomavirus, mainly genotypes 6 and 11. Furthermore, polymorphisms in *TAP1* appear to influence the selection of antigenic peptides and the transport process to the rough endoplasmic reticulum, for their subsequent presentation to T lymphocytes, an essential process against viral diseases and tumor processes. Previous studies have shown that individuals with those polymorphisms are susceptible to immune, infectious, and tumor‐related diseases. The present study aimed to determine the association between the *TAP1* rs1057141 (c.1177A>G) and rs1135216 (c.2090A>G) single nucleotide polymorphisms (SNPs) and RRP.

**Methods:**

A case–control study was carried out on a group of 70 individuals (35 controls and 35 patients). RRP diagnosis, HPV genotyping, and viral load were determined through histology and PCR. SNPs rs1057141 and rs1135216 were identified through allelic discrimination, using real‐time PCR. The haplotypic analyses were performed using the Arlequin 3.5 program.

**Results:**

HPV‐6 and HPV‐11 were the genotypes found in the samples. In the polymorphism analysis, rs1057141 showed no significant differences (*p* = 0.049, CI = 0.994–7.331). In contrast, a significant difference was found in rs1135216 (*p* = 0.039, OR = 2.4) in the allelic analysis, as well as in the dominant (*p* = 0.027, OR = 3.06), codominant (*p* = 0.033, OR = 3.06), and additive model (*p* = 0.043, OR = 2.505) in subjects with the G allele.

**Conclusion:**

The G allele in rs1135216 was associated with a genetic risk of susceptibility for RRP in a population in Western Mexico.

## Graphical abstract

The presence of single nucleotide polymorphism (SNPs) rs1057141 and rs1135216 in *TAP1* could influence the selection of antigenic peptides and their transport process into the rough endoplasmic reticulum for its subsequent presentation to T lymphocytes, an essential function in the defense against viral diseases and tumor processes. In the inheritance models analysis, in rs1135216, a significant difference was found (OR: 2.5), suggesting an association of risk for recurrent respiratory papillomatosis. In rs1057141, non‐association was found.

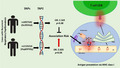

## 1 INTRODUCTION

Recurrent respiratory papillomatosis (RRP) is a respiratory tract disease that affects children and adults and is characterized by the recurrent proliferation of multiple papillomas. The proliferation of multiple papillomas within the airway in RRP primarily affects the larynx.^1^, ^2^ RRP is a potentially devastating and incurable disease, caused by human papillomavirus (HPV) infection. The HPV‐6 and HPV‐11 genotypes have been found to predominate in approximately 90% of cases.^3^, ^4^, ^5^ Few studies have been carried out on the epidemiology of RRP, and those conducted in the Americas have reported an incidence of RRP of 4.3/100,000 in children and 1.8/100,000 in adults.^5^, ^6^ RRP has not been widely studied in Mexico, resulting in a lack of incidence data. Furthermore, the intrinsic and environmental factors that influence the susceptibility for developing RRP and its clinical course have yet to be determined.

A dysfunctional immune response in patients with RRP has been reported. A low expression of the transporter associated with antigen processing (*TAP*) and major histocompatibility complex class I (*MHC‐I*) genes in benign papillomas has been found.^7^

The protein expression is inversely related to the frequency of disease recurrence.^7^, ^8^ A reduced TAP function has also been reported to decrease cell surface expression in MHC‐I molecules, which could be a strategy utilized by tumors and virus‐infected cells to escape immune surveillance.^9^ In addition, HPV has been posited to evade immune recognition by decreasing the expression of the major histocompatibility complex on the cell surface via decreased TAP expression.^10^

*TAP1* and *TAP2* are genes located in the major histocompatibility complex MHC class II region. They encode a non‐covalently associated heterodimer, which belongs to the ABC superfamily of transport proteins. TAP consists of two transmembrane domains that harbor the substrate‐binding site and two nucleotide‐binding domains that are responsible for ATP binding and hydrolysis. The TAP proteins are involved in the antigen presentation of endogenous antigens. They translocate peptides from the ubiquitin/proteasome pathway into the lumen of the endoplasmic reticulum (ER), which is essential for efficient loading of antigenic peptides onto the MHC‐I molecules. Therefore, the role of the TAP transport complex in adaptive immunity predetermines TAP as a target for infectious diseases and malignant disorders.^11^

*TAP1* gene alterations, such as polymorphisms, can produce a conformational change in the protein, including functional alterations.^11^ Various studies have reported different single nucleotide polymorphisms (SNPs) in the *TAP1* gene and their association with several immune diseases, viral infections, and even cancer development, mainly involving the rs1057141 and rs1135216 SNPs.^12^, ^13^, ^14^, ^15^ Those SNPs are located in the exon region and lead to changes in amino acids. Consequently, the amino acid changes could favor the susceptibility to HPV infection, as well as the development of severe cases of RRP.^7^, ^16^ Therefore, the present work aimed to determine the association between the *TAP1* gene polymorphisms (rs1057141, c.1177A>G, p.Ile393Val and rs1135216, c.2090A>G, p.Asp697Gly) and RRP.

## 2 MATERIALS AND METHODS

### 2.1 Recruitment of patients

Thirty‐five patients were recruited by convenience due to the low incidence of the pathology. Recruitment took place from 2015 to 2019 at two public hospitals in Western Mexico, the *Hospital Civil de Guadalajara Fray Antonio Alcalde* (*HCGFAA*) and the *Unidad Médica de Alta Especialidad*, *Centro Médico Nacional de Occidente* (*CMNO*). Patients were diagnosed with papillomatosis, employing flexible nasopharyngolaryngoscopy or a rigid endoscope. Biopsies for histopathologic and molecular diagnosis were taken during surgical resection. The Ethics Committee of the *Hospital de Especialidades*, *Centro Medico Nacional de Occidente* (*IMSS*) authorized the study, with approval no. 1031 R2014‐1301‐154.

### 2.2 Histopathologic stain

The tissue was processed for standard hematoxylin and eosin (H&E) sections.

### 2.3 Genomic DNA extraction from tissue

DNA extraction was performed for the screening and genotyping of HPV. 20–40 mg of papilloma tissue was subjected to digestion with tissue lysis buffer and proteinase K digestion, followed by binding to a silicon membrane and a series of washes in the presence of chaotropic salts, with the High Pure Kit DNA Preparation Kit (Roche), according to the manufacturer's instructions. The DNA obtained was then preserved at −80°C for later PCR analysis.

### 2.4 Genomic DNA extraction from blood

Ethylene diamine tetra‐acetic acid (EDTA) blood samples were collected from 35 unrelated control subjects and 35 patients with RRP. The extraction of genomic DNA was performed for the SNP genotyping. Samples were prepared, using the High Pure Kit DNA Preparation Kit (Roche). In brief, 200 μl of EDTA blood was subjected to proteinase K digestion in a lysis buffer, followed by binding to a silicon membrane and a series of washes in the presence of chaotropic salts. DNA was eluted from the membrane in 200 μl buffer and stored at −80°C until use.

### 2.5 Sample screening by polymerase chain reaction

To confirm the presence or absence of HPV in papilloma tissue, a *PCR* analysis was performed, utilizing 3 µl of DNA from tissue in 10 µl. The composition of the PCR mixture (10 µl) was 50 mM MgCl, 10 mM dNTP, and 5 U/ml HotStartTaq DNA polymerase (Qiagen). The reaction was subjected to 40 amplification cycles (4 min at 95°C, 1 min at 95°C, 1 min at 55°C, 1 min at 72°C) for the MY09 and MY11 primers, amplifying a 450‐bp fragment; 40 cycles of amplification (4 min at 95°C, 1 min at 95°C, 2 min at 40°C, 1 min at 72°C) for primers GP5^+^ and GP6^+^, amplifying a 143‐bp fragment, and 40 amplification cycles (2 min at 95°C, 30″ at 95°C, 30″ at 48°C, 1 min at 72°C) for primers L1C1 and L1C2, amplifying a 243‐262‐bp fragment, according to conditions previously reported by Qu et al^17^ and provided in the [Link], [Link]. One HPV positive control and one negative control were included in each PCR. Ten microliters of each PCR product were electrophoresed in a 1.5% agarose gel (Promega) and stained with ethidium bromide. The results are provided in the [Link], [Link].

*Genotype identification of HPV* Genotype identification and semi‐quantitative determinations of viral load were performed by Multiplex Real‐Time PCR, according to the manufacturer’s procedure, using the Anyplex™ HPV28 Detection Kit (Seegene™), with the CFX96 real‐time thermocycler™ (Bio‐Rad). Briefly, 5 µl of tissue DNA in 20 µl of reaction mixture was amplified with either primer set A or B. Real‐time PCR reactions were performed in two wells, with primer set A and B, identifying 19 high‐risk HPVs (HPV 16, 18, 26, 31, 33, 35, 39, 45, 51, 52, 53, 56, 58, 59, 66, 68, 69, 73, and 82) and nine low‐risk HPVs (HPV 11, 40, 42, 43, 44, 54, 6, 61, and 70), plus an internal control (human beta‐globin) in a single reaction. All reactions included positive and negative controls provided in the kit. In addition, knowledge of the step at which the melting curve became positive enabled the quantification of the viral load, from low (+; positive after 40 PCR cycles), to intermediate (++; positive within 31–39 cycles), to high (+++; positive before 31 cycles).^18^

### 2.6 Polymorphism (SNP) identification with allelic discrimination by real‐time PCR

Polymorphisms were identified using primers and FastStart Taqman™ hydrolysis probes for each of the SNPs in their wild‐type and polymorphic forms, using *TAP1* gene ID rs11352216, Cat # 531909_20 (1177A>G); *TAP1* rs1057141, Cat # 549926_20 (2090A>G) (Applied Biosystems™). The amplification reaction was carried out in a StepOne™ Thermal Cycler (Applied Biosystems™), under the following conditions: a pre‐incubation stage at 95°C for 600 s, then 40 cycles of 15 s each at 95°C, and 60 s at 60°C. A mixture was prepared for the reaction with FastStart TaqMan™ probe master 2×, SNP 20×, and genomic DNA from blood (20 ng/µl). Duplicate analyses were performed on 20% of the samples to avoid genotyping errors. For the analysis of the results, the StepOne™ software with the Allelic Discrimination Plot was used, which contrasts the normalized reporter dye fluorescence with the allele‐specific probes of the SNP assay.

### 2.7 Statistical analysis

The chi‐squared test was employed to analyze genotypic, haplotypic, and allelic frequencies, using the SPSS Statistics 23.0 program. The Hardy–Weinberg equilibrium, haplotype frequencies, and the estimated linkage disequilibrium were performed using the Arlequin 3.5 program. Finally, the odds ratio (OR) calculations were estimated with OpenEpi.com Statcalc, using a *p* < 0.05.

## 3 RESULTS

In the present study, 70 individuals (35 control subjects and 35 patients with RRP) were analyzed. The average age of the individuals with RRP was 25.13 years, with a range of 1–75 years (23 adults and 12 individuals in the pediatric age range [birth to 18 years of age]), and a higher percentage of men with RRP was observed (57.14%). The average age was 31.97 years in the controls, with a range of 19–54 years. The age distribution in the controls and cases was not significant (*p *= 0.100). In terms of sex distribution, there were 20 men (57.14%) and 15 women (42.86%) in the cases and 25 men (71.43%) and 10 women (28.57%) in the controls. After HPV screening and genotyping, the most frequent genotypes were HPV‐6 and HPV‐11. HPV‐6 was present in 21 of 35 patients, and HPV‐11 was present in only 14 patients. Of the 21 patients with HPV‐6, 15 presented with high viral loads (+++), whereas 6 presented with intermediate‐low viral loads (≤++). Five of the 14 patients with HPV‐11 had a high viral load (+++) and 9 had an intermediate‐low viral load (≤ ++).

The polymorphisms studied in the *TAP1* gene (rs1057141 [*p* = 0.973) and rs1135216 [*p* = 0.693]) were in the Hardy–Weinberg equilibrium. The comparison and distribution of the genotypic and allelic frequencies of each polymorphism are shown in Tables 1 and 2, respectively. The polymorphic homozygous genotype (GG) in the Asp697Gly (rs1135216) polymorphism was the most frequent, whereas the heterozygous genotype (AG) in the Ile393Val (rs1057141) polymorphism was the most frequent.

**Table 1:** Distribution and comparison of genotypic frequencies for both study groups

		*TAP1* rs1057141 (1177A>G)		*TAP1* rs1135216 (2090A>G)			
		AA	AG	GG	AA	AG	GG
Controls	n	24	10	1	26	8	1
	%	68.57	28.57	2.86	74.29	22.86	2.85
Total	35		35			35	
Cases	n	16	18	1	17	16	2
	%	45.71	51.43	2.86	48.57	45.71	5.72
Total			35			35	
*p*			0.176			0.171	

*p* < 0.05 was considered statistically significant. Controls: Control subjects. Cases: Patients with recurrent respiratory papillomatosis.

**Table 2:** *TAP1* allelic polymorphic frequencies for both study groups

Polymorphism	Cases		Controls		OR	CI 95%	*p*
	n	%	n	%			
1177A>G rs1057141							
A^a^	50	71.43	58	82.86		1	
G	20	28.57	12	17.14	1.933	0.860–4.344	0.107
Total	70	100	70	100			
2090A>G rs1135216							
A^a^	50	71.43	60	85.71		1	
G	20	28.57	10	14.29	**2.4**	**1.029**–**5.597**	**0.039**
Total	70	100	70	100			

Results highlighted in bold are statistically significant. *p* < 0.05 was considered statistically significant.

Abbreviations: CI, confidence interval; OR: odds ratio.

^a^ Reference category.

The distribution of the genotypic frequencies was evaluated through the models of genetic inheritance (Table 3). In the Asp697Gly (rs1135216) polymorphism, an increased risk was observed in the additive, in the dominant, and in the codominant model, due to an increment in the frequency of that polymorphism in patients with RRP.

**Table 3:** Analysis of the inheritance models of the SNPs (1177A> G, 2090A> G) in the *TAP1* gene in both study groups

SNPs	Genotype	Cases		Controls		OR	CI (95%)	*p*
		n	%	n	%			
1177A>G rs1057141								
Dominant	AA^a^	16	45.71	24	68.57		1	
	AG + GG	19	54.29	11	31.43	2.591	0.977–6.872	0.053
Recessive	AA + AG^a^	34	97.14	34	97.14		1	
	GG	1	2.86	1	2.86	1	0.060–16.649	1
Codominant	AA^a^	16	45.71	24	68.57		1	
	AG	18	51.43	10	28.57	**2.700**	**0.994**–**7.331**	**0.049**
	GG	1	2.86	1	2.86	1.500	0.087–25.754	0.779
Additive						2.169	0.890–5.285	0.088
2090A>G rs1135216								
Dominant	AA^a^	17	48.57	26	74.29		1	
	AG + GG	18	51.43	9	25.71	**3.059**	**1.117**–**8.373**	**0.027**
Recessive	AA + AG^a^	33	94.28	34	97.15		1	
	GG	2	5.72	1	2.85	2.061	0.178–23.827	0.555
Codominant	AA^a^	17	48.57	26	74.29		1	
	AG	16	45.71	8	22.86	**3.059**	**1.075**–**8.706**	**0.033**
	GG	2	5.72	1	2.85	3.059	0.257–36.421	0.752
Additive						**2.505**	**1.029**–**6.100**	**0.043**

Results highlighted in bold are statistically significant. *p* value was calculated by a chi‐squared test. *p* < 0.05 was considered statistically significant.

Abbreviations: CI: confidence interval; OR, odds ratio; SNPs: Single nucleotide polymorphism.

^a^ Reference category.

On the other hand, a linkage disequilibrium analysis at two sites (1177, 2090) was performed on the *TAP1* gene. Control subjects (*D*′ = 0.88, *r* ^2^ = 0.62) and RRP patients (*D*′ = 0.86, *r* ^2^ = 0.73) were in linkage disequilibrium, showing the inheritance pattern of the disease.

The haplotypes were subsequently established in 35 individuals with RRP and 35 control subjects, identifying the two polymorphic sites (rs1057141 and rs1135216). The allelic segregation phase was inferred probabilistically, with the maximum expected method, utilizing the Arlequin 3.5 program. Table 4 shows the frequencies of the haplotypes. In total, four haplotypes were identified in both study groups, and the most frequent haplotype was AA for both patients and controls (68.57% and 81.43%). Distribution was similar in the two study groups, and no significant differences were observed.

**Table 4:** Haplotypic frequencies of polymorphisms in the *TAP1* gene (1177A> G, 2090A> G) in patients with RRP and controls subjects

Haplotypes	Cases		Controls		OR	CI (95%)	*p*
	n	%	n	%			
AA^a^	48	68.57	57	81.43		1	
AG	2	2.86	1	1.43	2.375	0.209–27.005	0.473
GA	2	2.86	3	4.29	0.792	0.127–4.935	0.802
GG	18	25.71	9	12.86	2.375	0.978–5.769	0.052

*p* < 0.05 was considered statistically significant.

Abbreviations: CI: confidence interval; OR, odds ratio; RRP: recurrent respiratory papillomatosis.

^a^ Reference category, AA haplotype (1177A/2090A), AG haplotype (1177A/2090G), GA haplotype(1177G/2090A), GG haplotype (1177G/2090G).

## 4 DISCUSSION

TAP proteins play an essential role in the presentation of antigens in the MHC class 1 molecule. The variants in the *TAP1* and *TAP2* genes could lead to modifications in peptide translocation. Some mutations or polymorphisms in those genes could generate susceptibility to certain diseases,^12^, ^19^, ^20^, ^21^ including RRP. In that regard, the present study showed that polymorphisms in *TAP1* might be associated with the risk of developing RRP.

The 35 samples from patients with RRP were positive for HPV infection. The distribution of the HPV‐6 and HPV‐11 genotypes was similar to that reported in RRP samples from other studies^22^, ^23^, ^24^ The results of the present study also showed that HPV‐6 and HPV‐11 were the most frequent genotypes in individuals with RRP (68.7% and 60.8%, respectively), consistent with reports in 2017 from the National Institutes of Health (NIH) in the United States. The present study is one of the few reports analyzing the semi‐quantitative viral load of HPV.^25^, ^26^ In the samples, HPV‐6 was the genotype with the highest viral load (+++).

There are few Mexican studies on the frequencies of those polymorphisms and their association with RRP.^27^ In our study, we analyzed the rs1057141 (c.1177A>G, p.Ile393Val) and rs1135216 (c.2090A> G, p.Asp697Gly) polymorphisms in a population from the western region of the country diagnosed with RRP. The results showed that the distribution of frequency of the Ile393Val (rs1057141) and Asp697Gly (rs1135216) genotypes was the same for the cases and controls (*p* = 0.176 and *p* = 0.171, respectively), with wild homozygotes being more frequent in both polymorphisms (Table 1). The polymorphic allele frequency obtained in our study for the Ile393Val (rs1057141) polymorphism was similar in both the control and study groups (*p* = 0.107). In contrast, the allelic frequency for the Asp697Gly (rs1135216) polymorphism was significantly higher in the RRP patients (*p* = 0.039).

In the rs1135216 (c.2090A> G, p.Asp697Gly) polymorphism, a higher frequency of the homozygous polymorphic genotype was observed in RRP patients (5.72 in patients vs 2.85 in controls). In that regard, in the additive model, an OR = 2.505, 95% CI (1.029–6.1) *p* = 0.043 was obtained, indicating that having a copy of the G allele increases the risk 2.5 times. Therefore, homozygous individuals (GG) have twice the risk of heterozygous individuals. Also, in the dominant model an OR = 3.059, 96% CI (1.075–8706) *p* = 0.027 was observed. In the codominant model, the heterozygous genotype was significantly different (*p* = 0.033), with an OR = 3.059 and a 95% CI (1.075–8.706). Those findings are in agreement with previous studies associated with an increased risk of pulmonary tuberculosis (OR = 2.65 CI 95% = 1.78–3.96, *p* < 0.0001).^12^ In addition, Wang et al. reported that the rs1135216 and rs1067141 polymorphisms are associated with the risk of tuberculosis, nasopharyngeal carcinoma, and esophageal squamous cell carcinoma.^28^, ^29^

On the other hand, the rs1057141 polymorphism (c.1177A> G, p.Ile393Val) showed an increase in the heterozygous genotype frequencies of the RRP patients, compared with the control subjects (45.71 vs 22.86, respectively). In the codominant model, the heterozygous genotype was significantly different (*p* = 0.049), with an OR = 2.7 and a 95% CI (0.994–7.331). As a result, said genotype could produce a risk for the individuals that have it. However, the data showed no strong evidence of risk, probably due to the small sample size. Nonetheless, that polymorphism has been studied in other pathologies, and a meta‐analysis reported an increased risk of atopic disease in the allelic, dominant, and heterozygous model in the African population.^30^

In particular, the genetic basis of human disease is complicated. For example, given that many proteins are involved in the regulation of blood pressure, the combination of different mutations or polymorphisms located in various genes involved in the antigen presentation pathway has been proposed to be necessary for the development of hypertension.^31^

The fact that there are several polymorphisms in the *TAP1* gene, and the possibility of their being inherited together, made the haplotype analysis imperative. The four possible haplotypes of *TAP1* were detected in both groups. The AA haplotype (TAP*0101/02, 1A) was the most frequent (81.43% controls and 68.57% cases) and the GG haplotype (TAP*10401, 1D) occurred more frequently in the RRP patients (25.71% RRP vs 12.86% controls), but with no significant differences. The haplotype frequencies found in the present analysis are in accordance with those reported in the Mexican mestizo population from the highlands of Mexico and the Seri Indians of Sonora. In that study, the most frequent haplotypes were TAP*0101/02 and TAP*02011/12. The AA haplotype presented in 68.8% of the Seri Indians and in 75.8% of the mestizos, whereas the GG haplotype was reported in 31.2% of the Seri Indians and in 20.3% of the mestizo population.^27^

In summary, the findings of the present study suggest that the rs1135216 A/G polymorphism in *TAP1* is associated with a risk for RRP, and that there is a possible risk related to the rs1057141 A/G polymorphism in a western Mexican population. This is the first time that those genetic variants have been analyzed in RRP patients in a Mexican population, thus more studies conducted in other geographic areas and on other ethnicities are needed to support or contradict our results and conclusions.

The limitations of the present study include its sample size and cross‐sectional design. However, the results observed could set a precedent for other studies with larger populations. Due to the study design, it was not possible to evaluate disease severity or progression, and so longitudinal studies are needed to correlate the severity of RRP. In addition, the results of our study must be interpreted with caution due to the small sample size and the moderate statistical power, which could affect the reliability of the study’s outcome. Even though statistically significant differences between different genotypes were observed, the differences were so small that their physiologic significance is questionable.

## CONFLICT OF INTEREST

The authors declare that there is no conflict of interest.

## Supporting information

Figure S1

Table S1

## Data Availability

The data that support the findings of this study are available on request from the corresponding author. The data are not publicly available due to privacy or ethical restrictions.
