# Supplementary figures and images for: Association between the TAP1 gene polymorphisms and recurrent respiratory papillomatosis in patients from Western Mexico: A pilot study
Source: J Clin Lab Anal. 2021 Jan 28;35(4):e23712. doi: 10.1002/jcla.23712 (PMC8059727; doi:10.1002/jcla.23712)

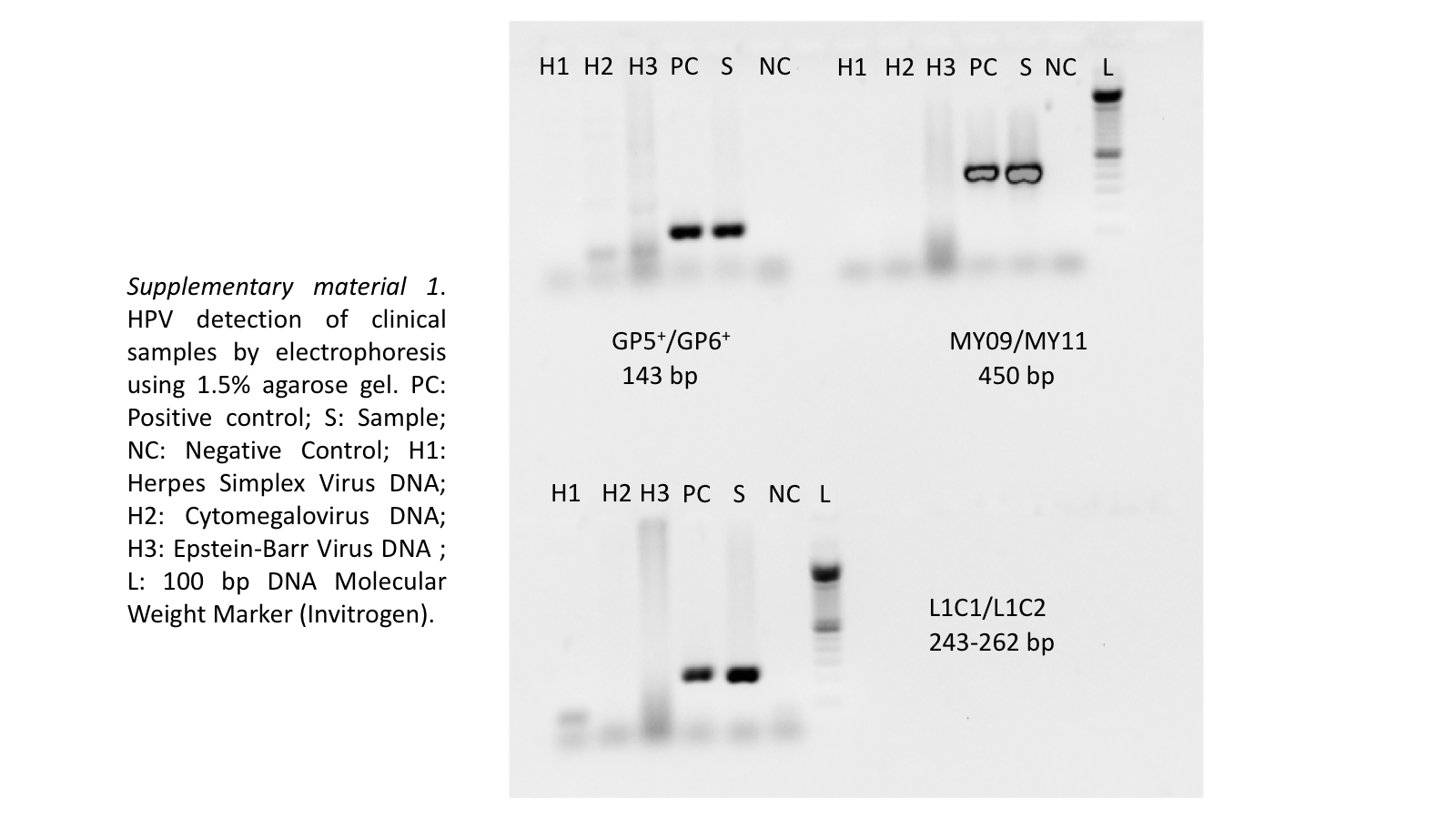

Supplement: Supplementary file 1 — Figure S1 [file JCLA-35-e23712-s002.tiff]
