# Supplementary material for: Association between the TAP1 gene polymorphisms and recurrent respiratory papillomatosis in patients from Western Mexico: A pilot study
Source: J Clin Lab Anal. 2021 Jan 28;35(4):e23712. doi: 10.1002/jcla.23712 (PMC8059727; doi:10.1002/jcla.23712)
Supplement: Supplementary file 2 — Table S1 [file JCLA-35-e23712-s001.docx]

**Supplementary material. Primers used in the study for HPV detection**

| **Primers** | **Primer Sequence (5’-3’)** | **PCR Conditions** | **Fragment size** |
| --- | --- | --- | --- |
| GP5+ | TTTGTTACTGTGGTAGATACTAC | | 94^o^C | 94^o^C | 40^o^C | 72^o^C | 72^o^C | | --- | --- | --- | --- | --- | | 4' | 1' | 2' | 1'30’’ | 4' | |  | 40 cycles | | |  | | 143 bp |
| GP6+ | GAAAAATAAACTGTAAATCATATTC |  |  |
| MY09 | CGTCCMARRGGAWACTGATC | | 95^o^C | 95^o^C | 55^o^C | 72^o^C | 72^o^C | | --- | --- | --- | --- | --- | | 4' | 1' | 1' | 1' | 7' | |  | 40 cycles | | |  | | 450 bp |
| MY11 | GCMCAGGGWCTATAAYAATGG |  |  |
| L1C1 | CGTAAACGTTTTCCCTATTTTTTT | | 94^o^C | 94^o^C | 48^o^C | 72^o^C | 72^o^C | | --- | --- | --- | --- | --- | | 2' | 30”' | 30”' | 1' | 7' | |  | 38 cycles | | |  | | 243–262 bp |
| L1C2 | TACCCTAAATACTCTGTATTG |  |  |

bp: Base pair; PCR: Polymerase chain reaction.
